# Exploring indicators of natural recovery from alcohol and drug use problems: findings from the life in recovery survey in Flanders

**DOI:** 10.1186/s13011-024-00604-y

**Published:** 2024-04-12

**Authors:** Florian De Meyer, Amine Zerrouk, Clara De Ruysscher, Wouter Vanderplasschen

**Affiliations:** Department of Special Needs Education, Ghent University, Henri-Dunantlaan 1, Ghent, 9000 Belgium

**Keywords:** Substance use disorder, Self-change, Spontaneous recovery, Treatment, Recovery capital

## Abstract

**Introduction:**

Research has established natural recovery (NR) as an important pathway to substance use recovery. Studies investigating correlates of NR have mainly focused on demographic and substance use variables rather than life circumstances. This study seeks to better understand the phenomenon of natural recovery by (i) validating the international scientific literature concerning demographic and substance use indicators of NR in Flanders and (ii) assessing the additional explanatory power of recovery strengths and barriers during active addiction, controlling for demographic and substance use covariates.

**Methods:**

A total of 343 persons in recovery from alcohol or drug use problems (≥ 3 months) completed an online cross-sectional survey in Flanders. Participants in NR and in recovery after following treatment were compared using multivariate linear regression models. Reasons for not following treatment were analyzed using inductive thematic analysis.

**Results:**

Higher education level, lower severity of dependence, and cannabis use as the main problem substance (vs. alcohol) were statistically significant (*p* < 0.05) correlates of NR. When scores for the number of barriers and strengths associated with active addiction were added, barriers (but not strengths) were significantly associated with NR. When barrier items were individually tested, having untreated emotional or mental health problems, having a driver’s license revoked and damaging property were statistically significant correlates. The most reported reason for not entering treatment was not experiencing any need to do so.

**Conclusion:**

The results highlight the importance of a holistic approach to recovery support across multiple life domains. Limitations and opportunities for further research are discussed.

## Introduction

Traditionally, approaches to addiction recovery have revolved around a clinical treatment model [1, 2]. Research has shown that recovery occurs through diverse treatment pathways, such as residential treatment, outpatient treatment, self-help groups, and less intensive forms of support [3–5]. Longstanding evidence suggests that a significant number of people recover from alcohol and drug use problems without engaging in formal treatment or participating in self-help groups, commonly referred to as natural recovery (NR) [6–8]. While estimates of NR vary and have predominantly focused on U.S. populations [6, 8–12], they consistently underscore the significance of NR as an important route to recovery. Recent representative studies have estimated that the prevalence of NR is approximately 50% of all persons who have resolved their alcohol and drug use problems [9] or were in remission from a DSM-5 alcohol use disorder [11].

Despite these prevalence estimates and a continuously growing body of research [6, 13, 14], knowledge about how persons in NR compare to persons in recovery following treatment is limited [15]. Comparative surveys between persons opting for treatment and persons in NR often focus on demographic and substance use profiles [15, 16]. Such studies have revealed that persons in NR are on average younger [9, 16–20], more frequently Caucasian [15, 19, 21], and have higher levels of education [15–17, 20, 22]. Regarding substance use history, research has shown that persons in NR exhibit lower severity of substance use problems [13, 18, 22–25], earlier onset of use [9], and shorter duration of dependence [15–17, 20]. Moreover, as many studies have focused on alcohol, Kelly and colleagues also highlighted the association between NR and cannabis as primary substance [9].

In light of the shift from an individual and pathology-oriented approach to a relational [17, 26, 27] and strengths-based [28] approach, addiction recovery research increasingly emphasizes the role of life circumstances [15] and diverse resources and barriers that are important in recovery processes. In this context, research into NR has been instrumental in the theoretical development of recovery capital [29]. Granfield and Cloud [30, 31] interviewed participants in NR and described how social capital facilitates NR. Notably, their study samples had considerable social stability that not only assisted in the creation and maintenance of social capital, but was reciprocally reinforced by it. Yet, other studies have suggested that despite differences between persons in NR and persons attending treatment, reasons for recovery and ways in which change is maintained are largely similar [13, 23, 32, 33].

Several international comparative studies have taken steps to identify differences between both groups, beyond demographic and substance use history variables. Although mostly based on convenience samples, a more extensive psychiatric history [2, 9, 17, 20, 22, 25], increased prevalence of childhood abuse and neglect [16, 17, 20], more extensive criminal history [9, 16, 17, 20], and more negative consequences of use [34] have been associated with following treatment. In contrast, a greater sense of coherence [16, 17, 20] and more reported social connectedness during active addiction [2] have been associated with NR.

Using a long-term recovery perspective, the Life in Recovery survey (LiR) has been set up in various countries to document diverse life circumstances across multiple domains before and after initiating recovery [2, 15, 35, 36]. In the U.S., Laudet and colleagues [15] compared treated and untreated persons in recovery and found that all assessed positive life experiences during active addiction were more common in the untreated group, while all negative life experiences were less frequent. However, these tests were univariate (chi-square) and did not control for demographic and substance use differences. Moreover, in contrast with natural recovery samples, the untreated group was conceptualized as not having followed formal treatment, and 85% reported 12-step group attendance. In Europe, the LiR has been applied in the UK, the Netherlands, and Belgium as part of the ‘Recovery Pathways’ project. Studies stemming from this project did not report on NR and only 4.6% of the participants included in this study were in NR [37].

Research concerning the relationship between life circumstances and NR is scarce, particularly in the European context. Existing survey research has focused primarily on demographic and substance use variables. A more comprehensive exploration of recovery strengths and barriers across multiple life domains considering demographic and substance use covariates can help to explain why some people follow treatment, while others recover without doing so. Starting from the international Life in Recovery survey [2, 15, 35, 36], this study aims to (i) validate the international scientific literature concerning demographic and substance use predictors of NR in Flanders and (ii) assess the additional explanatory power of strengths and barriers during active addiction for NR, controlling for demographic and substance use covariates.

## Methods

### Procedure

A convenience sample of people in recovery was recruited online through a social media campaign during the summer of 2022. We targeted persons in recovery from substance use problems who had diverse treatment experiences. The campaign was centered on Facebook and emphasized the importance of sharing personal recovery experiences. Collaborative efforts were made with several recovery support organizations in Flanders to disseminate the research on social media platforms, including treatment centers, peer recovery support groups, and social services. The eligibility criteria for participation were similar to those in previous Life in Recovery data collections. To be included, participants needed to (i) self-identify as being in recovery or having resolved a problem with alcohol and/or drugs for at least three months, (ii) be above 18 years of age, and (iii) have enough proficiency in Dutch to comprehend and respond to the survey questions. To address eligible participants not identifying with the ‘recovery’ concept [38], recruitment messages described potential participants both as ‘in recovery’ and as’ having had a substance use problem, but not anymore’. The social media campaign was carried out in two periods of 2 months each, with no financial reimbursement provided for participants. Responses were collected anonymously. All the respondents participated in this online cross-sectional study using Qualtrics. The study protocol was reviewed and approved by the medical ethical committee of the Ghent University Hospital, approval number BC-11704.

Of the 378 participants who filled out the survey, 35 were excluded because they reported over-the-counter medication as their biggest problem substance (*N* = 12), were less than three months in recovery (*N* = 17), or gave inconsistent responses (e.g., problem duration < 0 months, *N* = 6). ‘Without treatment’ was conceptualized in line with the criteria ofKelly and colleagues [9], meaning having no lifetime attendance in formal (residential and outpatient) and/or informal (self-help groups) support services specialized in remission from alcohol and drug use problems.

### Measures

The study applied the internationally administered Life in Recovery (LiR) survey, a 20-minute questionnaire about key life domains typically affected by addiction [35]. The LiR was developed through a literature review and insights from individuals in recovery to contribute to the understanding of the process of change from active addiction. Multiple assessments across different countries have consistently shown gains across a range of life domains in recovery, despite marked differences in, for example, the recovery pathways used [2, 39, 40]. We employed the version used in the European REC-PATH project in Flanders [3] and added three open-ended questions at the end of the survey about what recovery meant to participants. The survey included questions about sociodemographic characteristics, substance use, treatment history, followed by multiple questions about the participants’ life situation in the health, legal, financial, work/studies and social domain during active addiction, as well as in recovery.

The sociodemographic and substance use history questionnaire data included age, sex, place of residence, place of birth, education, marital status, age at first use, self-identified start of problem use, current use, and self-identified problem substances. To assess the severity of psychological substance use dependence, the Severity of Dependence Scale (SDS), a short 5-item scale with good psychometric qualities for alcohol and various types of drug dependence [41–43], was added. Participants completed this scale for each substance they experienced as a problem. The substances assessed were alcohol, cannabis, amphetamines/speed, cocaine, heroin, and methadone. Participants were able to add other substances they experienced as problematic to this list. The optimal cut-off scores for DSM-IV dependence reported in the literature typically range from 3 to 4 [44–46]. The Cronbach’s alpha for this scale in this sample was 0.740.

Participants were asked whether they ever engaged in a range of treatment modalities, including residential treatment, outpatient treatment, self-help group programs, or the use of online applications targeted at reducing or stopping substance use. Additional questions were asked about the timing, frequency, and perceived helpfulness (5-point Likert scale) of these types of treatment. Moreover, participants were asked if they ever received treatment for a psychiatric problem other than substance use.

Past positive and adverse experiences were retrospectively assessed through dichotomous items across multiple life domains, such as health, housing, finances, education, nutrition, sense of purpose, judicial involvement, meaningful activities, and employment [47]. LiR items were reclassified according to the Strengths and Barriers in Recovery scale (SABRS) into experienced strengths (15 items) and barriers (17 items). Participants were asked if each item applied to their situation during the peak of their addiction, as well as in recovery. Responses were coded as ‘1’ or ‘0’ based on the endorsement of the participant. This resulted in two composite scores [36, 48], developed to be proxy indicators of positive and negative recovery capital [36, 40] as conceptualized by Cloud and Granfield [29]. The internal consistency of the strengths was 0.742, and the internal consistency of the barriers was 0.735, indicating acceptable internal consistency.

To complement the quantitative comparison of persons in NR and recovery following treatment, we asked participants in NR to elucidate their reasons for not entering treatment or self-help groups.

### Data analysis

Statistical data analysis was conducted in R 4.3.1. Persons in the NR cohort were initially compared to persons in recovery after following treatment using Wilcoxon rank-sum, chi-squared, and Fisher exact tests. To address the first study objective, a multivariate logistic regression model was constructed based on seven commonly found demographic and substance use history indicators of NR. For the second objective, two variables were added to this multivariate model, namely, the SABRS composite score for (i) strengths and (ii) barriers experienced during active addiction. In a subsequent step, various multivariate logistic regression models were implemented to test the covariation of individual barrier items as defined by the SABRS and having followed treatment. These models incorporated individual barrier items while controlling for commonly found demographic and substance use variables. P-values were adjusted for false discovery rate following the Benjamini–Hochberg procedure. Multicollinearity was assessed using variance inflation factors for all models.

Answers to the open-ended question about reasons why participants in NR did not engage in treatment were coded thematically with NVIVO software to complement our quantitative results as well as previous research focusing on barriers to treatment [6, 14, 19, 49]. Responses were coded inductively [50] considering reasons for not following treatment, after which they were compared to identify common subthemes and overarching themes. Finally, we calculated the number of participants endorsing each theme and subtheme, to identify the occurrence of reasons for not entering treatment in this sample.

## Results

### Sample description

Table 1 describes the sample in terms of sociodemographic characteristics, substance use history variables, recovery phases, and SABRS scores. The sample consisted of 343 participants, 52 (15%) of whom reported NR. Groups were compared with Wilcoxon rank-sum tests, chi-square tests, and Fisher exact tests depending on the scale of the variables. P-values were adjusted for false discovery rate following the Benjamini–Hochberg procedure. After adjustment, statistically significant group differences (*p* < 0.05) were found for the reported main problem substance, SDS score, problem duration, age of first use, SABRS strengths, and SABRS barriers.

**Table 1:** Sample description

	Total sample (*n* = 343)	Recovery following treatment (*n* = 291)	Natural recovery (*n* = 52)	W, χ2 or P	df	Adjusted p-value
Age – mean (SD)	47.67 (12.13)	48.07 (12.17)	45.44 (11.78)	6558		0.18
Gender – n(%)				0.64	1	0.34
Male	185(54)	161 (55)	24 (46)			
Female	158 (46)	130 (45)	28 (54)			
Country of birth – n(%)				2.20	1	0.76
Belgium	330 (96)	279 (96)	51 (98)			
Other	13 (4)	12 (4)	1 (2)			
University/college – n (%)				4.59	1	0.05
Yes	141 (41)	112 (38)	29 (56)			
No	202 (59)	179 (62)	23 (44)			
Marital status – n (%)				8.11	3	0.13
Married/living together	152 (44)	126 (43)	26 (50)			
Divorced/separated	93 (27)	86 (30)	7 (13)			
Single	88 (26)	70 (24)	18 (35)			
Other	10 (3)	9 (3)	1 (2)			
Main substance – n (%)				14.27	2	0.006
Alcohol	228 (66)	203 (70)	25 (48)			
Cannabis	26 (08)	17 (6)	9 (17)			
Illicit drugs	89 (26)	71 (24)	18 (35)			
Multiple substances – n(%)				0.001	1	1
Yes	168 (49)	149 (49)	25 (48)			
No	175 (51)	152 (51)	27 (52)			
SDS-score – mean (SD)	9.61 (2.82)	9.95 (2.63)	7.75 (3.13)	4437		< 0.001
Problem-duration – mean (SD)	12.98 (9.30)	13.50 (9.04)	10.10 (10.26)	5393.5		0.004
Age of first use – mean (SD)	16.24 (5.68)	15.92 (4.74)	18.02 (5.75)	9260		0.02
Recovery phase – n (%)				4.65	2	0.20
Early recovery (< 1 year)	66 (19)	51 (18)	15 (29)			
Sustained recovery (1–5 years)	126 (37)	108 (37)	19 (37)			
Stable recovery (≥ 5 years)	151 (44)	132 (45)	18 (35)			
SABRS-strengths – mean (SD)	8.09 (3.10)	7.88 (3.12)	9.29 (2.70)	9537		0.007
SABRS-barriers – mean (SD)	5.85 (3.18)	6.22 (3.21)	3.83 (2.14)	4307.5		< 0.001

Participants had a mean age of 47.7 (SD = 12.1) years and almost all were born in Belgium. 41% of the total sample had a college or university degree. For participants in the NR, this was 56%. No sociodemographic group differences were found. Most participants (66%) cited alcohol as their primary problem substance, followed by cocaine (10%), amphetamines (9%) and cannabis (8%). Participants in NR, however, reported less (*p* < 0.05) alcohol (48% vs. 70%) and more cannabis (17% vs. 6%) and illicit drugs (35% vs. 24%) as the main problem substances than respondents who engaged in treatment. Half (49%) of the participants in the treatment group cited the use of multiple substances as problematic, as was the case for the NR group (48%). Participants in the NR group had a lower SDS score (7.75, SD = 3.13) than participants who followed treatment (9.95, SD = 2.63), and almost all reported a score higher than four (NR: 88%), as optimal cut-off scores for DSM-IV dependence mentioned in the analysis section typically range from 3 to 4 [44–46]. The mean reported duration of problem substance use was lower for participants in the NR group (10.10 years, SD = 10.26) than for participants following treatment (13.50 years, SD = 9.04). The mean recovery time of persons in NR was 4.89 years (SD = 6.51).

The most commonly followed treatment was outpatient treatment (82%), followed by residential treatment (63%), and self-help group attendance (63%), with most participants following a combination of support resources (74%). 58% of the total sample engaged in psychiatric treatment not focused on substance use (44% of those in NR, 60% of those in recovery following treatment). Two-thirds of the participants who reported alcohol as their main problem substance also engaged in self-help groups (66%), which was more than participants who reported cannabis (19%) or illicit drugs as their main concern (30%) (χ^2^ (2) = 46.29, *p* < 0.001).

### Logistic regression of NR with demographic and substance use variables

A first logistic regression model was set up consisting of multiple demographic and substance use indicators of NR. Table 2 presents the results of this model. Having a bachelor’s or master’s degree, SDS score, or cannabis as the main problem substance compared to alcohol were statistically significant (*p* < 0.05) when all other variables were constant.

**Table 2:** Model 1: regression model of NR based on demographic and substance use history

	B	SE	β	Standardized Odds Ratio^a^	CI (2.5-97.5%)		p-value
(Intercept)	-2.96	0.38		0.05	0.02–0.10		< 0.001
Age	-0.01	0.02	-0.16	0.85	0.56–1.27		0.43
Gender (female)	0.39	0.35		1.48	0.75–2.97		0.26
Higher education (yes)	0.88	0.35		2.42	1.24–4.83		0.01
SDS-score	-0.30	0.06	-0.86	0.42	0.30–0.59		< 0.001
Problem-duration	-0.02	0.02	-0.14	0.87	0.58–1.27		0.48
Age of first use	0.05	0.04	0.81	1.27	0.89–1.82		0.19
Main problem substance: cannabis	1.54	0.57		4.67	1.50-14.47		0.007
Main problem substance: other illicit drugs	0.70	0.50		2.02	0.76–5.28		0.15

^a^ Odds ratios were based on standardized coefficients of continuous variables (β) and dummy-coded categorical variables (B)

The Nagelkerke pseudo-R^2^ for this model was 0.26. A likelihood ratio test showed that this model explained NR better than a simpler model containing only age and gender, (χ^2^(6) = 51.02, *p* < 0.001).

### Logistic regression of NR including SABRS scores

Table 3 displays the results of adding the SABRS strength and barrier scores to Model 1. Age, SDS score, cannabis, and illicit drugs as the main problem substances compared to alcohol, and the SABRS barrier score were statistically significant (*p* < 0.05) in this model. When the number of barriers increased by one SD (3.18), the probability of NR decreased from 0.14 to 0.04. When the SDS score increased by one SD (2.82), the value decreased from 0.14 to 0.07. Compared to a probability of 0.05 when identifying alcohol as the main problem substance, this probability grew to 0.28 when cannabis was reported and 0.17 when illicit drugs were reported.

**Table 3:** Model 2: Logistic regression including SABRS strengths and barriers

	B	SE	β	Standardized Odds Ratio^a^	CI (2.5-97.5%)	p-value
(Intercept)	-3.29	0.43		0.04	0.02–0.08	< 0.001
Age	-0.04	0.02	-0.48	0.62	0.39–0.96	0.04
Gender (female)	0.18	0.38		1.19	0.57–2.53	0.64
Higher education (yes)	0.49	0.38		1.64	0.78–3.47	0.19
SDS-score	-0.24	0.07	-0.68	0.50	0.34–0.73	< 0.001
Problem-duration	0.003	0.02	0.03	1.03	0.67–1.53	0.90
Age of first use	0.05	0.04	0.23	1.25	0.84–1.89	0.27
Main problem substance: cannabis	2.03	0.62		7.62	2.26–26.49	0.001
Main problem substance: other illicit drugs	1.29	0.56		3.65	1.22–11.11	0.02
SABRS number of strengths	0.02	0.08	0.08	1.08	0.68–1.72	0.74
SABRS number of barriers	-0.42	0.10	-1.33	0.26	0.14–0.47	< 0.001

^a^ Odds ratios were based on standardized coefficients of continuous variables (β) and dummy-coded categorical variables (B)

The Nagelkerke pseudo-R^2^ for this model was 0.38. Likelihood ratio testing revealed that the model including SABRS scores was a better fit than the previous model without these scores (χ^2^(2) = 28.58, *p* < 0.001).

### SABRS barrier items and NR

Table 4 presents the results of the final regression analyses that explored individual SABRS barrier items by adding them to Model 1, i.e., controlling for age, gender, higher education, SDS score, problem duration, age of first use, and main substance problem. After adjusting for the false discovery rate with the Benjamini–Hochberg procedure, having untreated emotional or mental health problems, having a driver’s license revoked and damaging property were statistically significant (*p* < 0.05).

**Table 4:** SABRS barrier items resulting from multiple regression models

	B	Total prevalence	NR prevalence	Treatment prevalence	p-value	Adjusted p-value
Have untreated emotional or mental health problems	-1.32	0.73	0.52	0.82	< 0.001	0.01
Make regular visits to the emergency room (for other reasons than a medical or psychological issue you were already receiving treatment for)	-1.76	0.15	0.04	0.19	0.04	0.08
Regular use of health services	-0.15	0.23	0.21	0.25	0.73	0.78
Make use of tobacco products (E.g., cigarettes, shag, cigars or snuff)	-0.87	0.74	0.67	0.82	0.02	0.07
Have your driver’s license revoked	-1.73	0.29	0.11	0.35	0.002	0.02
Drive under the influence of alcohol or drugs	-0.69	0.73	0.71	0.79	0.06	0.12
Damage property of yourself or others	-1.21	0.41	0.23	0.48	0.003	0.02
Been arrested	-1.39	0.23	0.08	0.28	0.02	0.06
Been charged with a criminal offense	-2.53	0.22	0.04	0.27	0.02	0.06
Been to prison	-16.54	0.09	0	0.13	0.99	0.99
Were unable to pay the bills	-0.22	0.42	0.37	0.47	0.54	0.69
Have bad debts	-0.51	0.35	0.29	0.40	0.20	0.31
Regularly missed school or work	-0.34	0.41	0.33	0.45	0.38	0.53
Fired or suspended from work	-1.29	0.24	0.10	0.29	0.03	0.07
Dropped out of school or college	-0.62	0.29	0.21	0.33	0.17	0.30
Lose custody of children	-0.49	0.06	0.04	0.08	0.57	0.69
Experience family violence	-0.20	0.26	0.25	0.29	0.62	0.70

### Perceived reasons for not going to treatment

Table 5 summarizes the reported reasons by persons in NR for not entering treatment. The most common explanation of participants was that they perceived treatment as unnecessary (63%). This was explained in various ways. Some participants did not perceive their problem as severe enough (13%), others referred to their strong character (10%), changing life circumstances (8%), or other recovery support, enabling them to change without utilizing treatment services (6%). Also, multiple barriers to treatment utilization were reported (33%), of which perceived stigma was the most common (17%). Finally, multiple participants reported the will to prove that they could change themselves as an important reason for not entering treatment (9%).

**Table 5:** Reported reasons for not entering treatment (*n* = 52)

	n (%)
Treatment not necessary	33 (63)
The problem is not severe enough	7 (13)
Strong character	5 (10)
Changing and enabling life circumstances	4 (8)
Enough other recovery resources	3 (6)
Other	3 (6)
Treatment barriers	17 (33)
Stigma	9 (17)
Available treatment does not fit me	4 (8)
Bad experiences	3 (6)
Practical concerns (costs, waiting lists, time investment)	3 (6)
Lack of knowledge about available treatment opportunities	2 (4)
Motivation to self-change / prove to self	5 (10)

## Discussion

### Reprise of key findings

Based on this retrospective study with 343 participants in Flanders, we aimed to validate various demographic and substance use indicators of NR in a non-US population. The findings indicate a significant association between the number of barriers during addiction and NR, even after adjusting for demographic and substance use variables.

A first multivariate regression model was used to analyze the covariation of demographic and substance use variables with the recovery pathway followed. NR showed statistically significant covariation with three of the six hypothesized indicators: having followed higher education, severity of substance use problems (measured with the SDS) and cannabis as the main problem substance. In contrast to the results of the Wilcoxon rank-sum tests, duration of use and age of first use were not significant when the other variables in Model 1 were kept constant.

Model 2 included the number of reported SABRS strengths and barriers in addition to Model 1. In line with Laudet and colleagues [15], NR was related to more positive and less negative life experiences during addiction. However, when added simultaneously to the multivariate model, the number of experienced recovery barriers during active addiction (β=-1.33), but not the number of strengths was a statistically significant indicator of NR. In contrast to Model 1, reporting illicit drugs as the main problem substance was a significant indicator of NR, but having followed college or university education was not a significant indicator when SABRS scores were added to the model.

Third, we tested individual SABRS barrier items with a set of logistic regression models. These results are largely in line with life circumstance variables found in international literature. Having experienced mental health and emotional problems is a commonly accepted correlate of seeking treatment [2, 9, 17, 20, 22, 25]. These problems were assessed here as reported problems during active addiction [16, 17, 20, 22] rather than having received a mental health diagnosis [9] or having received treatment for psychiatric conditions before recovery [25]. Furthermore, and confirming the international literature, we identified additional legal recovery barriers covarying with NR [9, 17, 20]. Having a driver’s license revoked (but not driving intoxicated) or damaging property were statistically significant in our models after FDR adjustment, as were the raw p values, i.e., getting arrested and getting charged with a criminal offense. Both covariations can be explained by barriers to NR leading persons to search for treatment, as well as by the potentially helpful role of mental health and criminal justice mechanisms in stimulating people to seek treatment [9]. Finally, being fired or suspended at work was a significant indicator of NR before FDR adjustment in our model, as well as in a previous study by Carballo and colleagues [25]. While these authors interpreted this as a consequence of a less severe substance use history, the severity of dependence was controlled for in this study.

Fourth, all the coefficients of the SABRS barrier items were negative in this study (but not statistically significant at *p* < 0.05). The fact that the most common explanation for not following treatment was that participants in NR did not perceive treatment as necessary is particularly relevant here, as the results support the idea that persons in NR have less life circumstances that may hinder recovery during active addiction and hence, have more stable starting points for recovery.

Finally, despite observed differences indicating that persons in NR are less severely dependent and have more recovery resources during active addiction across the life domains tested, the sizes of the odds ratios found for demographic, substance use, and life circumstance variables were moderate. Several studies have already stressed similarities in what helps between recovery pathways [13, 23, 32, 33] and our results explain only partially who is in NR and who recovers after following treatment. It is important to stress the similarities between both groups and understand treatment as a continuum [4, 13] which is only one possible aspect of recovery, and happens in a multivariate life context [5, 13, 51]. Moreover, not needing treatment is only one explanation for NR, as barriers to treatment, such as stigma, play a significant role [6, 14, 49] and were commonly reported in this study. Conversely, the more positively formulated question as to why people have chosen or were urged to start treatment may be another important determinant which is not limited to treatment need.

### Limitations and suggestions for further research

Our findings affirm the covariation between multiple variables and NR and underscore the pivotal role of life circumstances across recovery journeys. However, this study has some limitations regarding the study design and instruments used. The cross-sectional design of this study precludes establishing causality and the composition of the sample, self-selected and recruited via social media, limits the generalization of results. Notably, research indicates that persons recruited through media (newspaper and radio/television) recruitment have a higher level of dependence [52], potentially contributing to the limited prevalence of NR in our self-selected sample and undervaluing recovery among persons with less severe dependence. Moreover, almost all participants were born in Belgium. The high prevalence of self-help group participation may be a type of recruitment bias resulting from the self-selected nature of this sample (i.e. AA members may be particularly motivated to answer such research calls because of their self-identification with recovery narratives and motivation to share their stories as part of their “service”). The greater self-help group engagement of participants who reported alcohol use as their main concern may be a result of the strong establishment of Alcoholics Anonymous in Flanders, and may explain the correlation between NR and illicit drug use (vs. alcohol) found in this sample. A larger and more representative NR sample may enable differentiation between NR participants (as mentioned above, the self-selected nature of our sample may have resulted in recruitment of participants with more severe histories of dependence) and open up opportunities for more intricate regression models, including interaction effects between variables.

Regarding the instruments used in this study, no diagnostic assessment was conducted beyond the SDS, limiting generalization of the findings. While the SDS has been validated as a proper assessment tool for alcohol and drug dependence [41–43] and scores across this sample were high on average (mean = 9.61), the validity of the SDS to distinguish dependence has been questioned in a sample of young adult, frequent cannabis users [53]. Furthermore, the utility of the SABRS for assessing recovery resources and barriers is acknowledged but constrained by its limitations in the type of items assessed as well as in the way of administration. Considering the former, SABRS items are mainly focused on life circumstances or what is commonly described as health and physical capital and may not sufficiently take into account the diverse dimensions of recovery capital [47, 54]. For example, known indicators of NR such as social capital [2, 19, 30, 55, 56] and sense of coherence [17] are not included in the scale. Qualitative research has elaborated on the social embeddedness of NR, despite participants’ often intrinsic attributions of natural recovery processes [19, 27, 30, 57]. Considering the latter, cross-sectional binary items preclude information about the degree of manifestation as well as time-bound interactions. Further quantitative and qualitative research should examine interactions between recovery resources across ecological domains in facilitating processes of NR. Despite the relevance of identifying differences between recovery pathways, research should step beyond differentiating between recovery pathways and focus on how persons and social environments with varying recovery resources can be supported through a continuity of formal, informal and community resources.

## Conclusion

A long-term recovery framework warrants the inclusion of life circumstances when comparing persons in NR with persons who followed treatment. This study validates several demographic and substance use indicators of NR commonly cited in scientific literature and points to the added explanatory value of experienced barriers across life domains. However, given the limitations of this cross-sectional retrospective study, further research is warranted to delve further into the temporal and relational dynamics shaping the influence of life circumstances on addiction and NR. Specifically, qualitative and longitudinal studies elucidating the interplay of life circumstances over time may help to contextualize these findings, given the dynamic, idiosyncratic [58], and relational [26, 27] nature of recovery processes across recovery pathways.

## Data Availability

All data generated or analyzed during this study are available from the corresponding author upon reasonable request.

## Abbreviations

DSM-IV: Diagnostic and Statistical Manual of Mental Disorders IV
FDR: False Discovery Rate
NR: Natural Recovery
LiR: Life in Recovery
SD: Standard Deviation
SDS: Severity of Dependence Scale
SABRS: Strengths and Barriers in Recovery Scale
